# Modelling the cost of place of birth: a pathway analysis

**DOI:** 10.1186/s12913-021-06810-9

**Published:** 2021-08-14

**Authors:** Vanessa L. Scarf, Serena Yu, Rosalie Viney, Seong Leang Cheah, Hannah Dahlen, David Sibbritt, Charlene Thornton, Sally Tracy, Caroline Homer

**Affiliations:** 1 Centre for Midwifery, Child and Family Health, Faculty of Health, University of Technology Sydney, PO Box 123 Broadway, Sydney, NSW 2007 Australia; 2 Centre for Health Economics Research and Evaluation, University of Technology Sydney, Sydney, Australia; 3 School of Nursing and Midwifery, Western Sydney University, Sydney, Australia; 4 University of Sydney, Sydney, Australia; 5 College of Nursing and Health Sciences, Flinders University, Adelaide, Australia; 6 Burnet Institute, Melbourne, Australia

**Keywords:** Economic analysis, Childbirth, Cost, Homebirth, Birth centre, Decision tree

## Abstract

**Background:**

In New South Wales (NSW), Australia there are three settings available for women at low risk of complications to give birth: home, birth centre and hospital. Between 2000 and 2012, 93.6% of babies were planned to be born in hospital, 6.0% in a birth centre and 0.4% at home. Availability of alternative birth settings is limited and the cost of providing birth at home or in a birth centre from the perspective of the health system is unknown.

**Objectives:**

The objective of this study was to model the cost of the trajectories of women who planned to give birth at home, in a birth centre or in a hospital from the public sector perspective.

**Methods:**

This was a population-based study using linked datasets from NSW, Australia. Women included met the following selection criteria: 37-41 completed weeks of pregnancy, spontaneous onset of labour, and singleton pregnancy at low risk of complications. We used a decision tree framework to depict the trajectories of these women and Australian Refined-Diagnosis Related Groups (AR-DRGs) were applied to each trajectory to estimate the cost of birth. A scenario analysis was undertaken to model the cost for 30 000 women in one year.

**Findings:**

496 387 women were included in the dataset. Twelve potential outcome pathways were identified and each pathway was costed using AR-DRGs. An overall cost was also calculated by place of birth: $AUD4802 for homebirth, $AUD4979 for a birth centre birth and $AUD5463 for a hospital birth.

**Conclusion:**

The findings from this study provides some clarity into the financial saving of offering more options to women seeking an alternative to giving birth in hospital. Given the relatively lower rates of complex intervention and neonatal outcomes associated with women at low risk of complications, we can assume the cost of providing them with homebirth and birth centre options could be cost-effective.

## Background

In New South Wales, Australia’s most populous state, there were 95 825 births to 94 449 mothers in 2017 [1]. Of these, 92.8% of women planned to give birth in a hospital, 6.3% planned birth in a birth centre, 0.25% of women planned a homebirth and the remaining 0.6% were born before arrival [1]. Maternity care in Australia is provided by the public and private sectors, with a 74% to 26% split respectively.

The evidence of the safety and benefits of birth at home or in a birth centre for women at low risk of complications is clear [2–5]. Access to these settings in New South Wales (NSW) and across Australia remains limited. There are 61 maternity services in NSW, 10 of which provide a birth centre option and three offer homebirth through a publicly funded model of care (where the midwives are employees of a maternity service) [6]. Most women who plan a homebirth, however, engage a privately practising midwife, at their own cost; these midwives are independent practitioners.

A hospital birth service, also referred to as a birth unit, birth suite, or labour ward, is staffed by midwives and doctors and provides maternity services to women with and without medical or obstetric risk factors. These birthing services are in both public and private hospitals. A birth centre offers women the option to give birth in a ‘homelike’ environment where the emphasis is on the physiological process of pregnancy and birth. Birth centres are staffed by midwives and are either located on the site of a maternity hospital (alongside birth centres) or in a location which may be on a hospital campus but does not offer obstetric and neonatal emergency care (freestanding birth centres). If a woman begins labour at a freestanding birth centre and develops a complication during the labour, she will be transferred to the nearest facility which provides higher level obstetric care. The ‘transfer’ process in an alongside birth centre is often a matter of re-locating a woman to a hospital birth room, most likely in the same building and often on the same floor as the birth centre. It is, however, an important distinction: if a woman planning to give birth in a birth centre develops a complication in labour, she is effectively transferred to higher level care in the hospital birth unit. Homebirth services are provided by midwives in private practice or by midwives employed by a health service and who work out of a maternity facility, known as a publicly funded homebirth model.

Anecdotally, it is asserted that offering homebirth or birth centre services is more costly to the health service despite few studies costing the place of birth in Australia. A study by Toohill et al. (2012) compared the cost of Midwifery Group Practice (MGP) and standard hospital care. MGP is a model of care which generally provides women continuity of midwifery carer, or group of carers and these midwives work across birth settings where available [7, 8]. Standard hospital care included hospital-based midwifery or obstetric care, or community-based General Practitioner (GP) shared care where the woman sees the GP for most of her antenatal consultations and has scheduled visits at the hospital where she plans to give birth. The majority of women in the MGP group gave birth in a birth centre. The results showed a cost saving overall for women in the MGP group compared with the hospital group applying a hospital-based costing system (AUD$4,696 vs $5,521) and (AUD$4,722 vs $5,641) when applying Australian Refined Diagnosis Related Groups (AR-DRGs) [8]. Similar results were found by Tracy et al, however the M@NGO study estimated costs related to model of care (continuity versus no continuity) rather than place of birth [9].

A systematic review of economic analyses of place of birth has shown a cost saving found for women giving birth at home or in a birth centre in eight of the eleven included studies, no difference in cost in two of the studies and a slight increase in one study which included initial set-up costs of a new birth centre [10]. An Australian micro-costing study [11] estimated the direct cost of vaginal birth for women planning birth at home, in a birth centre or in a hospital. The results revealed the overall costs were similar (AUD $2150, $2100 and $2097 respectively) however the services incurring the costs differed between homebirth and the other two settings. For women planning a homebirth, the majority of the cost was incurred by midwifery time and for women planning birth in a birth centre or hospital birth unit, facility overhead costs accounted for almost half the total cost [11].

A recent comparison of low-risk women choosing to give birth in a freestanding birth centre with a hospital obstetric unit in the United Kingdom (UK) estimated a saving of approximately £850 per woman [12]. Huynh et al. (2013) conducted a review of the cost of pregnancy in the United States of America (USA) to investigate the drivers of cost for payers in light of the increasing costs associated with pregnancy notwithstanding the decreasing birth rate. This review reported the varied results of the studies which included drivers such as inpatient care, pregnancy complications, pre- and post- term birth and pre-existing morbidity. The overall mean cost per hospital stay ranged from US$3,306 to US$9,234 however, costs associated with pre-term birth were as high as US$326,953 for an infant born at 25 weeks gestation [13]. The authors concluded that medical resource utilisation is increased, and therefore so are costs, with increasing complications during pregnancy. These findings are similar to those in an Australian study more than a decade ago estimating the cost of interventions in labour, which found the relative cost of birth increased by up to 50% for first-time mothers related to accumulating interventions [14]. Recent analyses of the costs by place of birth in NSW is lacking hence this study was undertaken.

The aim of this study is to estimate the cost of giving birth at home, in a birth centre or in a hospital for women at low risk of complications, by applying AR-DRG and other costs to each potential pathway identified in a decision tree developed using population-based data of pregnant women at low risk of complications in New South Wales.

## Methods

This study used a decision analytic modelling framework to construct a decision tree which illustrated the pathways of women at low risk of complications who gave birth in NSW between 2000 and 2012 [15]. The pathways were developed by identifying planned place of birth, and then using descriptive statistics, we determined each pathway including planned and actual place of birth, transfer to hospital labour ward, mode of birth and possible admission to neonatal care unit. Once the pathways were determined, an estimate of the cost of each pathway was applied to the terminal node by using Australian Refined Diagnosis Related Groups (AR-DRGs) (Table 1).

**Table 1:** AR-DRG definitions included in cost estimations

AR-DRG code^a^	Definition	Cost^b^
**O60C**	Vaginal delivery (minimal complications, singleton) - including women who had no intervention, or received any of the following: induction or augmentation of labour, epidural analgesia, narcotic pain relief, and/or minor perineal trauma.	**$4515**
**O60B**	Vaginal delivery (intermittent complications) - including women who had any of the following: multiple birth, instrumental vaginal birth with vacuum or forceps (not in operating theatre), post-partum haemorrhage (PPH), third or fourth degree perineal tear, episiotomy, or other ‘non-severe’ complications.	**$6108**
**O01C**	Uncomplicated Caesarean section, with or without labour.	**$9853**
**P68D**	Admission of neonate >= 37 weeks gestation, with minimal complications requiring observation for around 48 hours	**$4016**
**P68C**	Admission of neonate >= 37 weeks gestation, with intermediate complications requiring observation for 2-3 days	**$5562**

^a^Australian Refined Diagnosis Related Groups Version 5.2 Definitions Manual

^b^IHPA National Hospital Cost Data Collection Australian Public Hospitals 2016-17

### Data sources

We obtained linked data from the NSW Centre for Health Record Linkage (CHeReL) which linked data from the NSW Perinatal Data Collection (PDC), the NSW Admitted Patient Data Collection (APDC), the NSW Registry of Births, Deaths and Marriages (NSWRBDM) (death registrations only), and the Australian Bureau of Statistics (ABS) mortality data. We used these combined datasets to create a new dataset containing women who planned to give birth at home, in a birth centre or in a hospital, for the Birthplace in Australia Study [16] during the abovementioned years. The NSW Perinatal Data Collection (PDC) is a record of routinely collected data on all women who give birth in NSW, collected at the point of care (by midwives and doctors), most often through electronic medical record platforms. Maternal and infant data are collected on all livebirths and stillbirths greater than 20 weeks gestation or 400g birthweight (the Australian definition of viability) regardless of place of birth. The NSW APDC contains records of all NSW hospital inpatient separations (discharges, transfers, deaths) from public and private hospitals, public psychiatric hospitals, public nursing homes and private day procedure centres. Clinical data include identification and demographic data, International Classification of Diseases-Australian modification codes (ICD-10-AM) and procedure codes. The NSWRBDM is a permanent record of all registered births and deaths kept at the RBDM and the Australian Bureau of Statistics (ABS) compiles mortality data including primary cause and date of death.

### Population

Women were included if they were at low risk of complications, that is, 37 to 41 completed weeks gestation, pregnant with a single baby in the head down or ‘cephalic’ presentation. Women were also included if they had a spontaneous onset of labour (that is, no induction of labour) and were aged between 17 and 40 years (inclusive). Women who had an unplanned homebirth (born before arrival) or gave birth intentionally without a registered health provider present (free-birth) were not included in this cohort. The dataset itself includes data from both the public and private health sectors, however for the purposes of the cost modelling, a public sector perspective is taken.

Women were excluded if they experienced any obstetric or medical complication, mal-presentation (fetus in a position other than head-down), had a previous caesarean section, did not attend antenatal care or had their labour induced. Relevant variables and ICD-10-AM codes were identified from the PDC and APDC, a complex process which is described in full in Cheah et al. [16].

### Setting

This study expands on the investigation of the trajectories of women who plan to give birth at home, in a birth centre (both alongside and freestanding) or in a hospital [15]. Between 2000 and 2012, there were six alongside birth centres and three freestanding birth centres in NSW. The ‘transfer’ process from an alongside birth centre is often a matter of re-locating a woman to a hospital birth room, most likely in the same building and often on the same floor as the birth centre. It is, however, an important distinction: if a woman planning to give birth in a birth centre develops a complication in labour, she is effectively transferred to higher level care in the hospital labour ward. Homebirth services are provided by midwives in private practice or by midwives employed by a public health service and who work out of a maternity facility, known as a publicly funded homebirth model.

The public health service perspective is taken in this study. We received approval from the NSW Population and Health Services Research Ethics Committee, approval number HREC/14/CIPHS/15.

### Decision tree framework

**Fig. 1:**
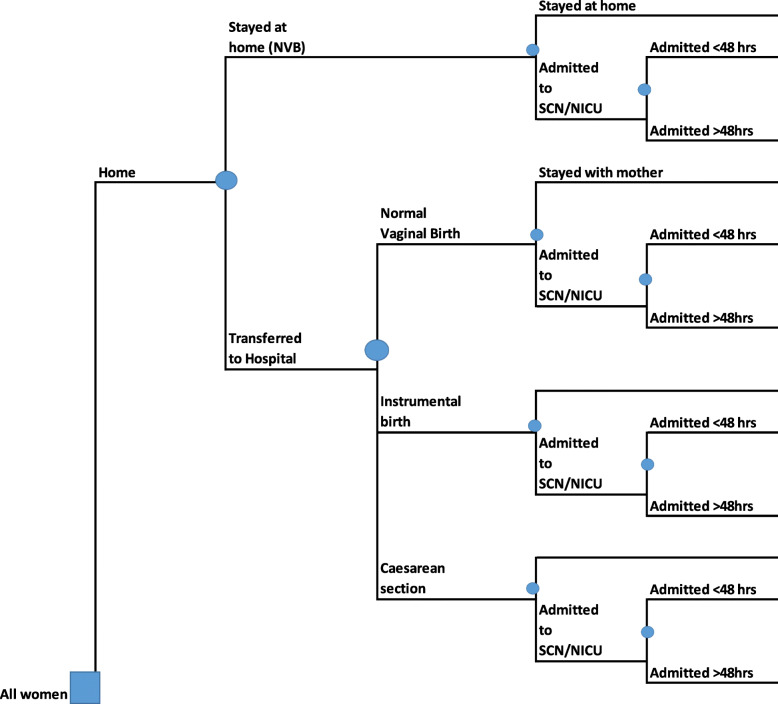
Decision tree framework

Decision analytic modelling provides a framework or structure that depicts the consequences of alternative options or treatments (and in this case, labour and birth outcomes) [17, 18]. The decision tree, interpreted from left to right, depicts the pathways of the women as their labour progressed, specifically noting transfer from home or a birth centre to a hospital, mode of birth (normal vaginal birth, instrumental birth- vacuum or forceps birth, and caesarean section) and admission to special care nursery/neonatal intensive care (SCN/NICU) for the baby. Figure 1 depicts the basic framework of the decision tree developed for this study. The decision node on the left represents the planned place of birth at the onset of labour. To the right of the decision node are chance nodes which represent the events that unfolded for the women and their infants. The branches which emanate from these chance nodes are mutually exclusive. The decision framework was chosen as it provides a visual structure which illustrates the pathways the women took using the linked dataset, and allows us to assign costs to each pathway.

### Pathway costs

Once the pathways were mapped in the decision tree, costs were allocated to each pathway. Included in the cost estimations were Australian Refined Diagnosis Related Group (AR-DRG) categories. AR-DRGs classify admitted patient episodes into groups with similar conditions and then match the resources required by the institution to provide the service [19]. The AR-DRGs associated with childbirth are in the major diagnostic category (MDC) 14: Pregnancy, childbirth and the puerperium (codes: O01A-O66B), the relevant codes are described in Table 1. Admission to the Special Care Nursery (SCN) / Neonatal Intensive Care (NICU) was also included, however, in the NSW Perinatal Data Collection, there is one variable which records admission to SCN/NICU, and does not distinguish between the two. In the cases where a baby was admitted to SCN/NICU, we were able to determine from the data if the admission was for greater than (or equal to) or less than 48 hours, and applied the corresponding AR-DRG. For simplicity, a baby who is not admitted to the ward (as is the case when the infant is healthy and under the full care of the mother) does not attract an AR-DRG and is thus costed at $0. This was assumed across the three birth settings for babies not admitted to the SCN/NICU.

To estimate the cost per woman, we calculated the total cost per pathway by multiplying the pathway cost with the number of women in each pathway group. We then added the totals of the pathways by place of birth and divided each total with the number of women in each planned place of birth. All costs are reported in Australian dollars (AUD). Table 2 contains the costs included in each pathway.

**Table 2:** Factors included in cost estimates

Planned place of birth	Mode of Birth AR DRG ($)	NICU admission AR DRG ($)	Total unit cost AUD
**Home**			
**Homebirth - SVB**	O60C (4515)	NA	$4515
**HB SVB + TF to NICU <48 hrs**	O60C (4515)	P68D (4016)	$8351
**HB SVB + TF to NICU >48 hrs**	O60C (4515)	P68C (5562)	$10077
**Mat TF + SVB**	O60C (4515)	NA	$4515
**Mat TF + SVB + NICU <48hrs**	O60C (4515)	P68D (4016)	$8351
**Mat TF + SVB + NICU >48hrs**	O60C (4515)	P68C (5562)	$10077
**Mat TF + IB**	O60B (6108)	NA	$6108
**Mat TF + IB + NICU <48hrs**	O60B (6108)	P68D (4016)	$10124
**Mat TF + IB + NICU >48hrs**	O60B (6108)	P68C (5562)	$11670
**Mat TF + CS**	O01C (9853)	NA	$9853
**Mat TF + CS + <48hrs**	O01C (9853)	P68D (4016)	$13869
**Mat TF + CS + >48hrs**	O01C (9853)	P68C (5562)	$15415
**Birth Centre**			
**BC SVB**	O60C (4515)	NA	$4515
**BC SVB + NICU <48hrs**	O60C (4515)	P68D (4016)	$8531
**BC SVB + NICU >48hrs**	O60C (4515)	P68C (5562)	$9851
**BC IB**	O60B (5562)	NA	$6108
**BC IB + NICU <48hrs**	O60B (5562)	P68D (4016)	$10124
**BC IB + NICU >48hrs**	O60B (5562)	P68C (5562)	$11670
**BC CS**	O01C (9853)	NA	$9853
**BC CS + NICU <48hrs**	O01C (9853)	P68D (4016)	$13869
**BC CS + NICU >48hrs**	O01C (9853)	P68C (5562)	$15415
**Hospital**			
**Hosp SVB**	O60C (4515)	NA	$4515
**Hosp SVB + NICU <48hrs**	O60C (4515)	P68D (4016)	$8531
**Hosp SVB + NICU >48hrs**	O60C (4515)	P68C (5562)	$9851
**Hosp IB**	O60B (5562)	NA	$6108
**Hosp IB + NICU <48hrs**	O60B (5562)	P68D (4016)	$10124
**Hosp IB + NICU >48hrs**	O60B (5562)	P68C (5562)	$11670
**Hosp CS**	O01C (9853)	NA	$9853
**Hosp CS + <48hrs**	O01C (9853)	P68D (4016)	$13869
**Hosp CS + >48hrs**	O01C (9853)	P68C (5562)	$15415

*Abbreviations*: *BC* birth centre, *CS* caesarean section, *HB* homebirth, *Hosp* hospital, *IB* instrumental birth (forceps, vacuum), *NICU* neonatal intensive care unit, *SVB* spontaneous vaginal birth, *TF* transfer

### Scenario analysis

In a scenario analysis, we recalculated the pathway costs and included antenatal consultation costs. The Independent Hospital Pricing Authority identified a national non-admitted cost per maternity patient of $2104 ($1550 allocated to antenatal care and $554 for postnatal care) [20] which we used to recalculate the cost per woman by place of birth.

Using the costs calculated including AR-DRGs and antenatal consultation costs, we proposed five different scenarios to model the cost of upscaling publicly funded homebirth and birth centre options. *Scenario 1* estimates the total cost to the health service using the current proportions of 0.4% of women planning a homebirth (current rate in NSW), 6% planning a birth centre birth and 93.6% planning a hospital birth. For *Scenario 2,* we calculated the cost of birth in these settings if the proportions were increased to 1% homebirth, 9% birth centre birth and decreased to 90% hospital birth. *Scenario 3* is a calculation of the costs of birth in the three settings if these services were up-scaled similar to maternity services in the United Kingdom, that is 2.5% homebirth, 5% birth centre and 92.5% hospital obstetric unit [21]. For *Scenario 4* the cost of upscaling homebirth to 1% and birth in a birth centre to 15% were calculated and Scenario 5 calculated the upscaling of homebirth to 2.5% and 15% in a birth centre. We calculated the total cost of these scenarios for a population of 30,000 women. This is the estimated number of childbearing women in NSW who meet the criteria of low-risk pregnancy and spontaneous onset of labour per year.

## Results

### Planned place of birth

There were 496 387 women identified as meeting the criteria for inclusion (Table 3). Of these, 0.4% planned a homebirth, 6.0% planned a birth centre birth and 93.6% planned birth in a hospital. There were differences in the demographic characteristics across the three birth settings. Women planning a homebirth were older (mean 31.7 years, standard deviation (SD) 4.7) compared with women who planned birth in a birth centre (mean 29.1 years, SD 5.1) or in a hospital (mean 28.9, SD 5.3). There was a higher proportion of women having their first baby (nulliparous women) in the hospital and birth centre groups (45.1% and 42.7% respectively) compared to the homebirth group (29.9%). We included women who were at term (37 to 41 completed weeks gestation) and who went into spontaneous labour. Overall, the highest proportion of women laboured at or beyond 40 weeks, with 67.1% in the homebirth group, 57.1% planning a birth centre birth and 54.0% planning a hospital birth.

**Table 3:** Demographic characteristics

	Hospital n = 464,630 (%)	Birth Centre n = 29,933(%)	Home n = 1824 (%)
**Maternal age (Years) Mean (SD)**	28.9 (5.3)	29.7 (5.1)	31.7 (4.7)
**<20**	20,733 (4.5)	767 (2.6)	19 (1.0)
**20-24**	81,183 (17.1)	4189 (14.0)	118 (6.2)
**25-29**	142,161 (30.0)	9110 (30.4)	439 (23.2)
**30-34**	147,523 (31.1)	10,271 (34.3)	700 (37.0)
**35-39**	68,094 (14.4)	5251 (17.5)	504 (26.7)
**>40**	4936 (1.1)	345 (1.2)	111 (5.9)
**Previous pregnancies (>20 weeks)**			
**0**	209,664 (45.1)	12,782 (42.7)	546 (29.9)
**1**	150,364 (32.4)	10,727 (35.8)	662 (36.3)
**2**	65,633 (14.1)	4460 (14.9)	373 (20.4)
**> 3**	38,969 (8.4)	1964 (6.6)	243 (13.3)
**Gestation (weeks) Mean (SD)**	39.5 (1.04)	39.6 (1.04)	39.7 (1.02)
**37**	22,518 (4.8)	1073 (3.6)	66 (3.6)
**38**	62,166 (13.4)	3231 (10.8)	163 (8.9)
**39**	129,050 (27.8)	7930 (26.5)	370 (20.3)
**40**	185,175 (39.9)	11,558 (38.6)	821 (45.0)
**41**	65,721 (14.1)	6141 (20.5)	404 (22.1)

### Pathway costs of place of birth

**Fig. 2:**
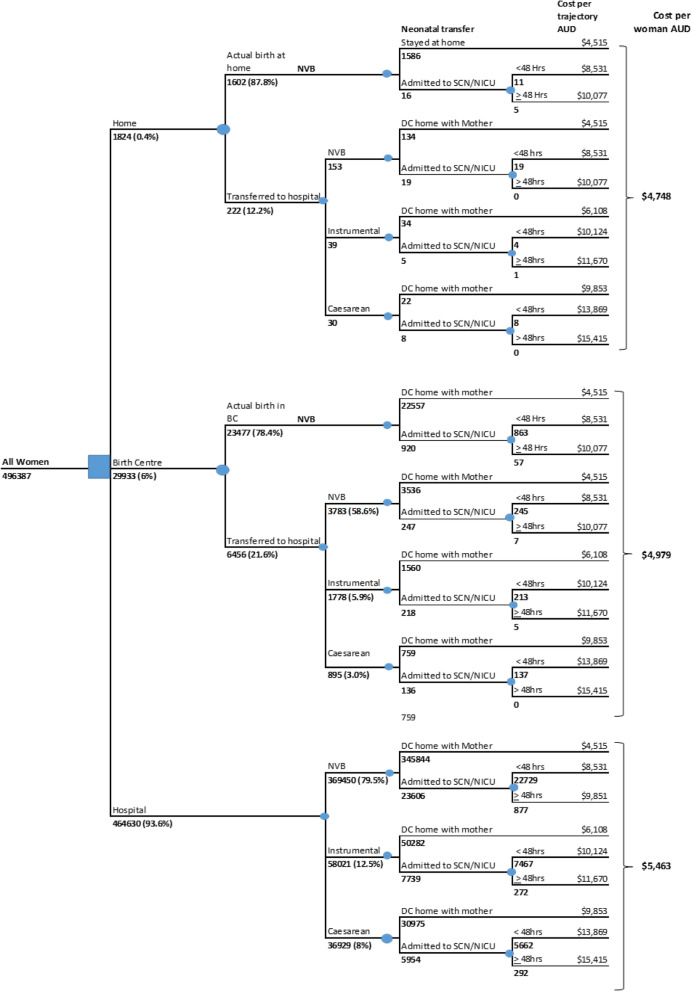
Pathway costs and mean costs of birth setting

The women planning birth at home or in a birth centre had twelve potential outcome pathways. The women planning a hospital birth have the most direct pathway, differing only by mode of birth and neonatal outcome. Women in the planned birth centre and homebirth group differed by transfer and then mode of birth and neonatal outcome. Figure 2 illustrates these potential pathways and the number of women in the sample who followed each pathway are presented below each branch. A description of the conditional probabilities of each pathway has been presented in a previous publication [15]. Briefly, the normal vaginal birth rate in women planning a homebirth was 96.2% (including women who transferred to hospital), 91.1% for women planning birth in a birth centre (including transfers) and 79.5% in the hospital birth group. The transfer rate from home or a birth centre to hospital was 12.2% and 21.5% respectively. Instrumental birth rates for the three settings were 2.1% (homebirth), 5.9% (Birth Centre) and 12.5% (hospital), and caesarean sections occurred in 1.6% of planned homebirths, 3.0% of planned birth centre births and 7.9% of births planned in hospital.

Each pathway accrued a cost (Table 2) depending on the resources used. In Fig. 2, for example, a woman planning a homebirth who is transferred to hospital for an instrumental birth and whose baby is well enough to be discharged home with her incurred a cost of $6524. A woman planning a birth centre birth or a hospital birth with the same outcome incurred a cost of $6108. In these three pathways the AR-DRG was the same (O60B), and the difference in the cost is attributable to the cost of transfer by ambulance (see Table 2). Another example is the pathway illustrating a caesarean section (O01C) and neonatal admission to the special care nursery/neonatal intensive care unit for over 48 hours (P68C). For a woman planning a homebirth who is transferred to hospital and receives these interventions, the estimated cost was $15 831. The same pathway for a woman planning a hospital birth incurs a cost of $15 415. Again, the difference in cost relates to transfer costs. Finally, the estimated cost per women (Fig. 2) by place of birth was $484 more costly in the hospital group compared with the birth centre, $715 more costly in the hospital group compared with homebirth and $231 more costly in the birth centre compared with homebirth.

### Proposed scenarios

The following scenarios calculate the total cost to the public health system for 30 000 women in NSW by place of birth when AR-DRGs only are used and when AR-DRGs plus an estimated cost of antenatal care is included (Table 4).

**Table 4:** Modelling cost by place of birth per year in NSW

N=30000	Proportion	AR DRG only	Estimated AN care and AR DRG
**Scenario 1: Current proportions**			
**Home**	0.004	$569,760	$826,560
**Birth Centre**	0.06	$8,962,200	$12,814,200
**Hospital**	0.936	$153,401,040	$213,492,240
**Total**	1	$162,933,000	$227,133,000
**Scenario 2: Upscaling to 1% homebirth and 9% Birth Centre**			
**Home**	0.01	$1,424,400	$2,066,400
**Birth Centre**	0.09	$13,443,300	$19,221,300
**Hospital**	0.9	$147,501,000	$205,281,000
**Total**	1	$162,368,700	$226,568,700
**Difference**^a^			-$564,300
**Scenario 3: Upscaling 2.5% homebirth 5% birth centre (similar to UK proportions)**			
**Home**	0.025	$3,561,000	$5,179,500
**Birth Centre**	0.05	$7,468,500	$10,624,500
**Hospital**	0.925	$151,598,250	$210,983,250
**Total**	1	$162,627,750	$226,827,750
**Difference**^a^			-$305,250
**Scenario 4: Upscaling to 1% homebirth and 15% birth centre**			
**Home**	0.01	$1,424,400	$2,066,400
**Birth Centre**	0.15	$22,405,500	$30,996,000
**Hospital**	0.84	$137,667,600	$191,595,600
**Total**	1	$161,497,500	$224,658,000
**Difference**^a^			-$2,475,000
**Scenario 5: Upscaling to 2.5% homebirth and 15% birth centre**			
**Home**	0.025	$3,561,000	$5,179,500
**Birth Centre**	0.15	$22,405,500	$30,996,000
**Hospital**	0.825	$135,209,250	$188,174,250
**Total**	1	$161,175,750	$224,349,750
**Difference**^a^			-$2,783,250

^a^Difference between the total of the scenario compared to Scenario 1

Scenario 1 estimated the total cost to the health service for a cohort of 30,000 women in NSW per year using the current proportions of women planning birth at home, in a birth centre and in a hospital. The average cost per place of birth was calculated to be $4748 for homebirth, $4979 for birth in a birth centre and $5463 for planned hospital births (Fig. 2). When the estimated cost of antenatal care is included, the cost increases by $2104, resulting in a total cost of birth at home, in a birth centre and in a hospital of $826,560, $12,814,200 and $213,492,240 respectively.

In scenario 2, we recalculated the costs the three places of birth increasing the proportions of planned births to 1% at home, 9% in a birth centre and 90% in a hospital. When antenatal costs are included, the total cost saving per year was $564,300, reducing the total expenditure by 0.25% when compared to the costs associated with the current proportions of 0.4% homebirth, 6% birth centre and 93.6% hospital birth (Scenario 1).

Scenario 3 estimates the costs when homebirth and birth centre services are increased to 2.5% and 5% respectively, as is the case in the UK. The total saving to the health service per year amounts to $305,250 when antenatal costs are included, when compared to the current proportions.

We further tested the scaling up of homebirth and birth centre services to 1% and 15% in scenario 4 and 2.5% and 15% in scenario 5 and calculated an annual cost saving of $2,475,000 and $2,783,250 respectively. These scenarios amounted to a saving of over 1%.

## Discussion

This is the first study to examine cost by place of birth using standardised cost weights, that is, AR-DRGs. This approach was taken to more closely reflect the cost to the health system, as the estimates and scenarios are based on actual and proposed numbers of women coming through a publicly funded maternity system. We found differences in the cost per woman by place of birth which can be attributable largely to mode of birth. During the development of the NSW dataset, we endeavoured to create a cohort as similar as possible however we recognise that there would be unobservable characteristics in the women included which may influence the results. Our selection processes enabled us to identify women with key characteristics which place them closely aligned, specifically, spontaneous onset of labour, cephalic presentation, 37-41 completed weeks gestation (at term), with no documented pre-existing medical or pregnancy complication [16]. The greatest proportion of women who attracted the AR-DRG with the lowest value (O60C) were in the homebirth group (96.2%) followed by 91.1% in the birth centre group and 74.4% in the hospital group.

The impact of the complex outcomes for women in all groups contributed to the incremental increase in cost from homebirth to birth centre to hospital. For women planning a homebirth for example, the proportion of neonates admitted to NICU/SCN was 2.3% (<48hrs) and 0.3% (>48hrs) which attracts a cost of between $8947 and $15831 depending on the mode of birth. Neonates of women planning birth in a birth centre had an SCN/NICU admission rate of 4.9% (<48hrs) and 0.46% (>48hrs) in the hospital birth group, the neonatal admission rates to SCN/NICU were 7.7% (<48hrs) and 0.3% (>48hrs) with costs of between $8531 and $15415 again, depending on the mode of birth and no addition of transfer cost.

The national costing authority in Australia, the Independent Hospital Pricing Authority (IHPA) found that non-admitted (antenatal and postnatal) care was similar across most childbearing women with the exception of women with very complex pregnancies. The cost of the admitted birth episode (and in the case of a homebirth, the “admission” relates to the birth episode at home/ transfer to hospital) differed significantly as the driver for that cost was mode of birth indicating that significant savings can be made by “clinically warranted reductions in the rate of interventions during birth” ([20] p24). Research has shown significant differences in modes of birth related to birth setting, including increased spontaneous vaginal birth rates for women planning birth at home or in a birth centre [21, 22]. This translates to a lower cost per birth when comparing birth setting [12, 23, 24]. There are countries, however, which employ very few DRG categories to cost childbirth. In a study by Or et al (2012) of European countries, the variation of DRG-related birth codes ranged from three in Austria and Poland (where the payment for vaginal birth and caesarean section were the same) to seven in England and eight in Germany describing several birth complications [25]. This has the potential to provide a perverse incentive to service providers to be more prone to intervention during birth to increase funding from government [26, 27].

When we proposed an up-scaling of services to enable women to plan a birth at home or in a birth centre, the cost to the public health service resulted in a slight decrease in cost over a 12-month period. While the increase in homebirth options were considerable comparatively (scenarios 2 and 4 represented a 250% increase and scenarios 3 and 5 were a 625% increase in homebirth) the proportions remained very small. Considering the absolute increase of services was modest, it would be feasible to offer a greater number of women options including publicly supported homebirth and birth centre care while utilising the existing infrastructure. There may be additional costs related to training and accreditation of staff and facilities, which would ultimately be recouped over time with the prospected decrease in intervention. A limitation of proposing this increase in service options is that there exists only anecdotal reports of the demand by women to enter into a program which offers an alternative to hospital birth; reports of waiting lists cannot be quantified and further research into the apparent demand is warranted.

### Strengths and limitations

This study represented the provision of homebirth services in a publicly-funded model however, in NSW, more than half of homebirths were attended by midwives in private practice. Smooth transfers to hospital require a networked or integrated service. Additionally, transfer costs were not included in the total cost for women who transferred to hospital from home as not all transfers occur via ambulance. If an ambulance was required, we calculated an additional $416 for transfer assuming a ten-kilometre distance from the nearest maternity facility^1^. In countries where different birth setting options are integrated in to the health system, for example the United Kingdom, New Zealand or the Netherlands, the decision for women about where they will give birth is more contemporaneous, and the transfer processes are well understood and facilitated by the health services [28–30].). In Australia, homebirth is uncommon and integration into the health services varies across individual services, as do attitudes relating to the acceptability and demand among midwives and obstetricians [31, 32]. Fox et al (2018) explored the processes and interactions that occurred during transfer from home to hospital during a birth for both women and health professionals. They found the divergence of philosophical beliefs related to safety and risk negatively influenced their understanding and respect for the women and the midwives who were attending their birth. This resulted in an “us and them” dynamic which created an atmosphere of conflict rather than collaboration in some transfer cases [33]. The cost of transfer also varies with the distance from the maternity facility, which may increase (or decrease) the cost of transfer from home or a freestanding birth centre.

## Conclusion

The findings from this study offer some clarity into the financial saving of offering greater options to women seeking an alternative to giving birth in hospital. Maternity service provision is complex and admission for intrapartum care drives the costs related to overheads, interventions and outcomes. Given the relatively lower rates of complex intervention and neonatal outcomes associated with women at low risk of complications, we can assume the cost of providing them with homebirth and birth centre options could be cost-effective.

## Data Availability

The data that support the findings of this study are not available. It is a condition of the agreement between the Centre for Health Record Linkage (CHeReL) and the researchers that the dataset remain confidential. We are not permitted to make any part of the linked data available to any party outside those named on the research team who have been granted access.

## Abbreviations

ABS: Australian Bureau of Statistics
APDC: Admitted Patient Data Collection
AR-DRG: Australian Refined-Diagnosis Related Groups
AUD: Australian dollars
CHeReL: Centre for Health Record Linkage
GP: General Practitioner
ICD-10-AM: International Classification of Diseases-Australian modification
MDC: Major Diagnostic Category
MGP: Midwifery Group Practice
NICU: Neonatal Intensive Care
NSW: New South Wales
NSWRBDM: NSW Registry of Births, Deaths and Marriages
PDC: Perinatal Data Collection
SCN: Special Care Nursery
UK: United Kingdom
USA: United States of America

## Footnotes

1 Transfer costs were calculated using the latest fees and charges for the NSW Ambulance Service which includes a callout fee (AUD$382) and rate per kilometre (AUD$3.44). The final transfer cost was calculated at AUD$416.40 which assumes a ten-kilometre distance to the nearest maternity hospital.
